# Dissecting the Immune Stimulation Promoted by CSF-470 Vaccine Plus Adjuvants in Cutaneous Melanoma Patients: Long Term Antitumor Immunity and Short Term Release of Acute Inflammatory Reactants

**DOI:** 10.3389/fimmu.2018.02531

**Published:** 2018-11-02

**Authors:** María B. Pampena, Holliday C. Cartar, Gerardo Rubén Cueto, Estrella M. Levy, Paula A. Blanco, María M. Barrio, José Mordoh

**Affiliations:** ^1^Centro de Investigaciones Oncológicas-Fundación Cáncer, Buenos Aires, Argentina; ^2^Grupo de Bioestadística Aplicada, Departamento de Ecología, Genética y Evolución, Instituto de Ecología, Genética y Evolución de Buenos Aires (IEGEBA-UBA/CONICET), Facultad de Ciencias Exactas y Naturales, Universidad de Buenos Aires, Buenos Aires, Argentina; ^3^Department of Biotherapy, Instituto Alexander Fleming, Buenos Aires, Argentina; ^4^Fundación Instituto Leloir, IIBBA-CONICET, Buenos Aires, Argentina

**Keywords:** cutaneous melanoma, CSF-470 allogeneic cell vaccine, C-reactive protein, innate immunity, adaptive immunity

## Abstract

As cutaneous melanoma (CM) currently remains with a bleak prognosis, thorough investigation of new treatment options are of utmost relevance. In the phase II/III randomized clinical trial (CASVAC-0401), the repeated immunization of stages IIB-III CM patients with the irradiated, allogeneic cellular CSF-470 vaccine plus the adjuvants bacillus Calmette-Guerin (BCG) and recombinant human granulocyte macrophage colony-stimulating factor (rhGM-CSF) demonstrated a significant benefit over IFN-alpha2B treatment in distant metastasis-free survival. Here we present on the short and long term immune monitoring results after completing the 2-year protocol; a continuation of the previous report by Mordoh et al. (1). We demonstrate that the repeated CSF-470 vaccinations stimulated a long term cellular and humoral immunity response directed against the vaccine antigens. In the case of 2 patients, we are able to show that a similar immune response was generated against autologous antigens. Evaluation of inhibitory receptor co-expression on patient's T cells indicates that the vaccination protocol did not stimulate T cell exhaustion. In order to better understand the basis for the efficacious vaccine responses observed, we investigated the short term immune events following vaccine injection. A significant increase in C-reactive protein (CRP) and IL-6 was observed 24 h after vaccination, with *in vitro* studies suggesting IL-6 production occurs in the vaccine site. We demonstrate that CRP enhances the cytotoxicity of peripheral blood mononuclear cells (PBMC) against melanoma cells in an *in vitro* model. Additionally, CRP stimulates the release of pro and anti-inflammatory cytokines from PBMC. As our results demonstrate that successive vaccinations with CSF-470 plus adjuvants promoted an increase in both anti-tumor innate and adaptive immunity, we propose a subsequent model of action.

## Introduction

Cutaneous melanoma (CM) is responsible of most skin cancer deaths (2). Although recent therapeutic advances based in targeted therapies for BRAF-mutated CM and of anti-PD1 monoclonal antibodies (MAbs) have changed the somber prognosis of metastatic CM (3), the challenges are still great. With respect to adjuvant therapies in stage III patients, it has been reported that mutated BRAF inhibitors and anti-PD-1 MAbs increase relapse-free survival, although follow-up is short and about two thirds of patients at risk of recurrence are unprotected (4, 5). Since CM is an immunogenic tumor, most probably due to its high mutation rate (6), the use of vaccines as immunogens have been assayed for a long time. One of several approaches has been to use single antigens (Ags) expressed in CM, such as cancer-testis Ags, in vaccine formulations. However, a recent clinical study of recombinant Mage-3 Ag in adjuvancy in 1344 stage III CM patients showed no benefit in disease-free survival or overall survival (7). Based on the idea that a single Ag-based vaccine may permit escape from immune response precisely due to the high mutation rate of CM, we have used multi-Ag allogeneic vaccines to foster immunity against commonly shared melanocytic differentiation Ags and cancer testis Ags, expecting that epitope spreading might be a mechanism of further including private Ags in the immune response.

In a previously reported phase II randomized clinical trial, stages IIB, IIC, and III CM patients were treated for 2 years in a 2:1 ratio with the CSF-470 vaccine plus bacillus Calmette-Guerin (BCG) and recombinant human granulocyte macrophage colony-stimulating factor (rhGM-CSF) vs. medium-dose IFN-alpha2B (α2B), and a superiority in distant metastasis-free survival for the vaccine arm was observed (1). In that study, cellular and humoral immunity against tumor Ags was reported at 6 months after starting vaccination.

Exhausted T cell responses have been documented following numerous viral infections as a result of Ag persistence which causes a decrease in T cell functionality and promotes a particular T cell phenotype exhibiting co-expression of inhibitory receptors (8). Furthermore, T cell exhaustion has also been described following adenovirus vector immunization in a murine model (9). It was therefore important to determine if repeated vaccinations with CSF-470 vaccine plus BCG plus rhGM-CSF over the 2-year treatment led to continued T and B cell stimulation or to exhaustion. In order to better understand the basis of the efficacious vaccine responses observed in vaccinated patients, we explored the short-term systemic reactions to vaccination with CSF-470 plus BCG plus rhGM-CSF, which had not been studied until now. We demonstrate that repeated vaccinations maintain or even increase the adaptive immune response, and that each vaccination induces a short-term release of acute phase proteins and cytokines which probably trigger innate immune responses further supporting the adaptive response.

## Materials and methods

### CSF-470 vaccine & CASVAC-0401 study design and treatment

The **CASVAC-0401** (ClinicalTrials.gov: NCT01729663) phase II clinical study was approved by the Ethics Committee of the Instituto Alexander Fleming (Buenos Aires, Argentina), and by the Argentine Regulatory Agency (ANMAT), and it was conducted according to the Declaration of Helsinki Principles. All patients signed informed consent. This study investigated the CSF-470 vaccine in combination with BCG and rhGM-CSF vs. medium-dose INF-α2B as adjuvant treatments in post-surgery CM patients stages IIB, IIC, and III (1). The CSF-470 vaccine is composed of 4 lethally-irradiated CM cell lines (MEL-XY1, MEL-XY2, MEL-XY3, and MEL-XX4). All cell lines were established in house from metastatic CM tumors and were cultured as previously described (10). Throughout the 2 year trial, patients in the vaccine arm should have received 13 vaccinations and had peripheral blood samples obtained at baseline (PRE), and after 6 (P1), 12 (P2), and 25 (P3) months for immune monitoring; P1 sample was obtained 2 months after the 5th vaccination; P2 sample was obtained 2 months after the 8th vaccination and P3 sample was obtained 1 month after the 13th vaccine administration. Peripheral blood mononuclear cells (PBMC) were purified using a Ficoll density gradient (GE Healthcare, UK) from 200 ml of peripheral blood collected at each time-point (with heparin anti-coagulant), and kept cryopreserved in freezing medium consisting of 40% Albumin (UNC Hemoderivados, Argentina), 50% Dulbecco's Modified Eagle's Medium (Gibco, ThermoFisher, USA) and 10% Dimethyl Sulfoxide (Merck, USA) (1 × 10^7^ cells per cryotube) until use. Serum was collected from 50 ml peripheral blood samples (without heparin) at every time-point and kept frozen at −80°C until use.

In a subset of 7 patients included in an extended vaccination protocol, for which patients signed informed consent, distinct serum samples were obtained from 7 ml blood drawn immediately before (pre-vaccination) and 24 h after vaccination in order to evaluate the short-term systemic response to vaccination. As follow-up visits allowed for multiple samples from selected patients, a total of 12 samples were used for the short-term immune assessments.

### Monitoring of cellular immune response

#### IFN-γ enzyme-linked immunospot assay (ELISPOT)

Sufficient quantities of PBMC could be recovered from 10 patients. Thawed PRE, P1, P2, and P3 patients' PBMC from cryotubes frozen with 1 × 10^7^ cells, were allowed to rest for 2 Hs at 37°C and counted using a Neubauer chamber and an inverted microscope (Olympus, Japan), recovering a median cell yield of 7 × 10^6^ and viability of 80%. Cells were then seeded (1 × 10^6^) in 1 mL of Complete Medium consisting of RPMI 1640 (Invitrogen, USA) supplemented with 10% heat-inactivated human AB sera, 2 mM glutamine, 100 U/mL penicillin, 100 μg/mL streptomycin, 2.5 mM HEPES and 50 U/mL of IL-2 (Laboratorio Pablo Cassará SRL, Argentina), in 24-well plates (Costar, Corning, USA). PBMC were stimulated with CSF-470 lysate in a 3:1 ratio (Ag-presenting cells from PBMC population: lysed CSF-470 cells), and cultured at 37°C, in 5% CO_2_ for 12 days. For 2 patients (#006 & #026) in which it was possible to obtain autologous tumor cells from metastases which had developed after (#006) or during (#026) treatment, cellular responses were also measured against their own tumor cell lysates. Every 3 days, fresh complete medium with IL-2 was added to the cultures. The CSF-470 lysate and autologous tumor lysates were obtained by 5 cycles of freezing (liquid nitrogen, 3 min) and thawing (7 min, 37°C) followed by centrifugation at 1,000 g for 10 min to separate nuclei. Lysate aliquots were frozen at −80°C until use.

MultiScreen-IP opaque 96-well plates (High Protein Binding Immobilon-P membrane, Millipore, Bedford, MA, USA) were coated overnight at room temperature with 100 μl/well of 5 μg/ml mouse anti-human IFN-γ mAb (BD Biosciences, USA) in 1X PBS (Gibco). Then, the plates were washed and blocked for 2 Hs at room temperature with 200 μl/well of culture medium consisting of RPMI 1640 (Invitrogen) supplemented with 10% heat-inactivated fetal bovine sera (FBS, Gibco), 2 mM glutamine, 100 U/mL penicillin, 100 μg/ml streptomycin, and 2.5 mM HEPES. Harvested PBMC from the 12-day cultures of patients' PRE and the 3 different POST time point samples stimulated with lysate were collected and added to the plates in duplicate at 2.5 × 10^5^ cells/well and cultured with either culture medium alone or, as a positive control, with the addition of the monoclonal antibody against human CD3 OKT-3 (30 ng/ml, BD Pharmingen, USA) plus PHA (1/1,000, M form, Gibco) for 24 h at 37°C, 5% CO_2_. The plate was washed twice with deionized water and then thrice with PBS−0.05% Tween-20. A total of 1,000 μl/well of 2 μg/ml anti-human IFN-γ-biotinylated mAb (BD Pharmingen) in 1X PBS/10% FBS were added, and the plates were incubated for 2 Hs at room temperature. After 3 washes with PBS−0.05% Tween-20, the plate was incubated with 50 μl/well of streptavidin HRP (BD Pharmingen) diluted 1:100 in PBS 1X/10% heat-inactivated FBS for 1 H at room temperature. The plates were then washed 4 times with PBS−0.05% Tween-20 and then twice with PBS. Spots were visualized by adding 50 μl/well of AEC Substrate (BD Biosciences) for 2 min. Substrate reaction was stopped by washing the plate with deionized water. Plates were scanned using an AID iSPOT ELR088IFL analyzer and AID Elispot reader software 7.0 (AID, Germany) was used to quantify the number of spots per well. Spots could be properly quantified from 1 to 350 spots/well due to saturation of the signal. The positive controls consisted of the OKT-3 and PHA re-stimulated wells, and subtracted background signal was obtained from wells containing no cells and culture medium only.

#### FACs analysis of T cell exhaustion markers

Patient's PBMC were incubated with anti-human MAbs: APC-H7-CD3 (clone SK7), PerCP-Cy5.5-CD4 (clone RPA-T4), Pe-Cy7-CD8 (clone RPA-T8), APC- PD-1 (clone MIH4), BB515-TIM-3 (clone TD3) and BV421- CTLA-4 (clone BNI3) (BD Biosciences). Lymphocytes were gated in FSC/SSC dot plot (≥30,000 events), CD4^+^ and CD8^+^ T cells were gated within CD3^+^ cells. Isotype-matched irrelevant MAbs were used as negative controls. All samples were acquired on a BD FACSCanto using FACSDiva software (BD Biosciences) and analyzed with FlowJo 10.0.7 software (USA).

### Monitoring of humoral immune response

#### Serum antibodies analysis by ELISA

Sufficient quantities of serum could be recovered from 13 patients. Humoral responses were measured against live melanoma cell lines that compose CSF-470 vaccine (MEL-XY1, MEL-XY2, MEL-XY3, and MEL-XX4). For 2 patients (#006 & #026) in which it was possible to obtain autologous tumor cells from metastases which developed after the start of treatment, humoral responses were also measured against their own tumor cells. CM cells, either allogeneic or autologous tumor cells, were seeded in 96-well culture plates (10^4^ cells/well) and cultured for 48 Hs. Reactivity was detected by ELISA in PRE, P1, P2, and P3 serum samples (1/10, 1/100, and 1/1,000 dilutions)—in 4 replicates—using peroxidase-conjugated goat anti-human IgG (Abcam, UK) and o-phenylenediamine as substrate (450 nm). A healthy donor (HD) serum sample was used to set the background value.

#### Serum cytokines analysis by ELISA

IL-6, TNF-α, IL-1β, and IL-10 cytokines were measured in patient sera in triplicate using ELISA kits following manufacturer's instructions (BD Biosciences).

#### C-reactive protein analysis (CRP)

CRP concentration was measured in patient sera and in supernatants from *in vitro* cocultures of vaccine components plus PBMC and fibroblasts using the CRP assay on the ARCHITECT System following manufacturer's instructions (Abbott, USA) in Alexander Fleming Institute Laboratory of Clinical Analysis and Molecular Diagnosis (Buenos Aires, Argentina).

### IL-6 release by cell cocultures

A total of 5 × 10^5^ PBMCs purified from HD were cultured in 1 mL RPMI 1640 medium supplemented with 10% heat-inactivated FBS, 2 mM glutamine, 100 U/ml penicillin, and 100 μg/ml streptomycin together with 5 × 10^5^ CSF-470 vaccine cells with or without adjuvants (160,000 colony forming units—CFU—of BCG and 10 μg/ml rhGM-CSF), in 24-well plates. The cocultures were incubated at 37°C 5% CO_2_ for 120 Hs within which every 24 h the media was collected and centrifuged at 1,500 rpm for 5 min to retrieve supernatants to be stored at a −80°C until the measurement of IL-6 through ELISA (BD Biosciences).

Monocytes were purified from PBMCs using a CD14 positive magnetic selection (Miltenyi Biotec, Germany), with 90–95% of purity assessed by FACs. Lymphocyte population was recovered from the CD14 negative population. Both cell populations were cultured separately in 1 mL RPMI 1640 medium supplemented with 10% heat-inactivated FBS, 2 mM glutamine, 100 U/mL penicillin, and 100 μg/mL streptomycin, with or without adjuvants (160,000 CFUs of BCG plus 10 μg/mL rhGM-CSF). After 24 h incubation the medium was harvested and centrifuged at 1,500 rpm for 5 min. Supernatants were collected and stored at −80°C until IL-6 measurement through ELISA kit (BD Biosciences) as described.

### *In vitro* CRP effect

#### To evaluate the effect of CRP on cytokine release from PBMCs

PBMCs from HD were cultured (5 × 10^5^/ml) for 24 h with a low (2 μg/ml) and a high (20 μg/ml) concentration of CRP (Sigma-Aldrich, USA) in RPMI 1640 medium (Invitrogen) supplemented with 10% heat-inactivated FBS (Gibco), 2 mM glutamine, 100 U/ml penicillin, and 100 μg/ml streptomycin. After 24 h the medium was collected, centrifuged at 1,500 rpm and supernatants were stored at −80°C until further analysis. Concentrations of cytokines TNF-α, IL-1β, IL-6, and IL-10 were measured in supernatants using ELISA kits following the manufacturer's instructions (BD Biosciences).

#### To evaluate the effect of CRP on PBMC cytotoxicity toward target melanoma cells

To model *in situ* metastases' site *in vitro*, WI-38 fibroblast cells were cultured and harvested by trypsinization such that 5 × 10^5^ cells per well were allowed to adhere in 6-well plates overnight. In 1:1 proportion, cultured MEL-XY3 cells were added to the fibroblast monolayers. PBMCs collected from HD were separated by a density gradient and added to the wells in a 5:1 ratio of effector to target MEL-XY3 cells with or without 20 μg/ml CRP and/or 100 pg/ml IL-6. The concentrations of CRP and IL-6 were based on values seen in patient short-term sera samples. The control included fibroblasts, MEL-XY3, and PBMC in the absence of CRP and IL-6. All cocultures were incubated in melanoma medium (Dulbecco's Modified Eagle Medium: nutrient Mixture F12 (1:1) supplemented with 2 mM glutamine, 20 nM sodium selenite, 100 μM ascorbic acid, 0.3 mg/ml galactose, 0.15 mg/ml sodium pyruvate and 5 μg/ml insulin, 100 IU/ml penicillin, 10 μg/ml streptomycin, 2 μg/ml ciprofloxacin, and supplemented with 10% heat-inactivated FBS (Natocor, Argentina). After 24 h, all cells were collected using trypsin for detachment, washed, and re-suspended in a 2X concentrated culture medium with 0.7% sterilized agarose at a cell density of 2.5 × 10^4^ MEL-XY3 cells/ml. Suspensions of 7,500 MEL-XY3 cells were then plated over a 1% agar underlayer, immediately observed through an inverted microscope to ensure adequate cell suspension, and then incubated for a duration of 14 days with fresh medium supplementation every 3–4 days. After 14 days, all wells were fixed with formaldehyde and photos were taken using an inverted microscope (Olympus). Colonies were considered to be >30 cell aggregates and were counted using ImageJ. Three independent experiments were done with 3 different HD, and triplicates were done for each condition.

### Statistics

Statistical analysis were performed using Infostat 2017 software (11) and Graphpad Prism 7.0 software. The significance level was set as *p* < 0.05.

For immunomonitoring analysis (PRE, P1, P2, P3), generalized linear mixed models (GLMMs), with a binomial error distribution and logit link function was used (12). A random effect “patient” was added to account for the non-independence among observations made on the same patient. An observation-level random effects was added to absorb the extra-Binomial variation in the data (13). The fixed effect was time. *Post-hoc* comparison was done with DGC multiple comparison test (14).

For IL-6 release analysis, data were analyzed by fitting general linear mixed-effects models with a normal error distribution, considering time, treatments and their interactions as fixed factors, and HD as a random factor. The model was tested for homoscedasticity and normality of residuals by visual assessment of plots. Since homoscedasticity was not accomplished, the model was fitted by the addition of the VarIdent variance structure (12) to treatment and time. Besides, a first order autoregressive correlation structure was added to account for the non-independence of repeated observations of the same HD. *Post-hoc* comparison analysis was done with DGC multiple-comparison test.

For serum cytokines and CRP analysis, paired *T*-test or Wilcoxon matched-pairs signed rank test was used.

For PBMC cytotoxicity experiments, GLMM was used, with a poisson error distribution. A random effect HD was added to account for the non-independence among observations made on the same HD. The fixed effect was time. *Post-hoc* comparison was done with DGC multiple comparison test.

## Results

### Long term cellular and humoral immune responses

**Figure 1:**
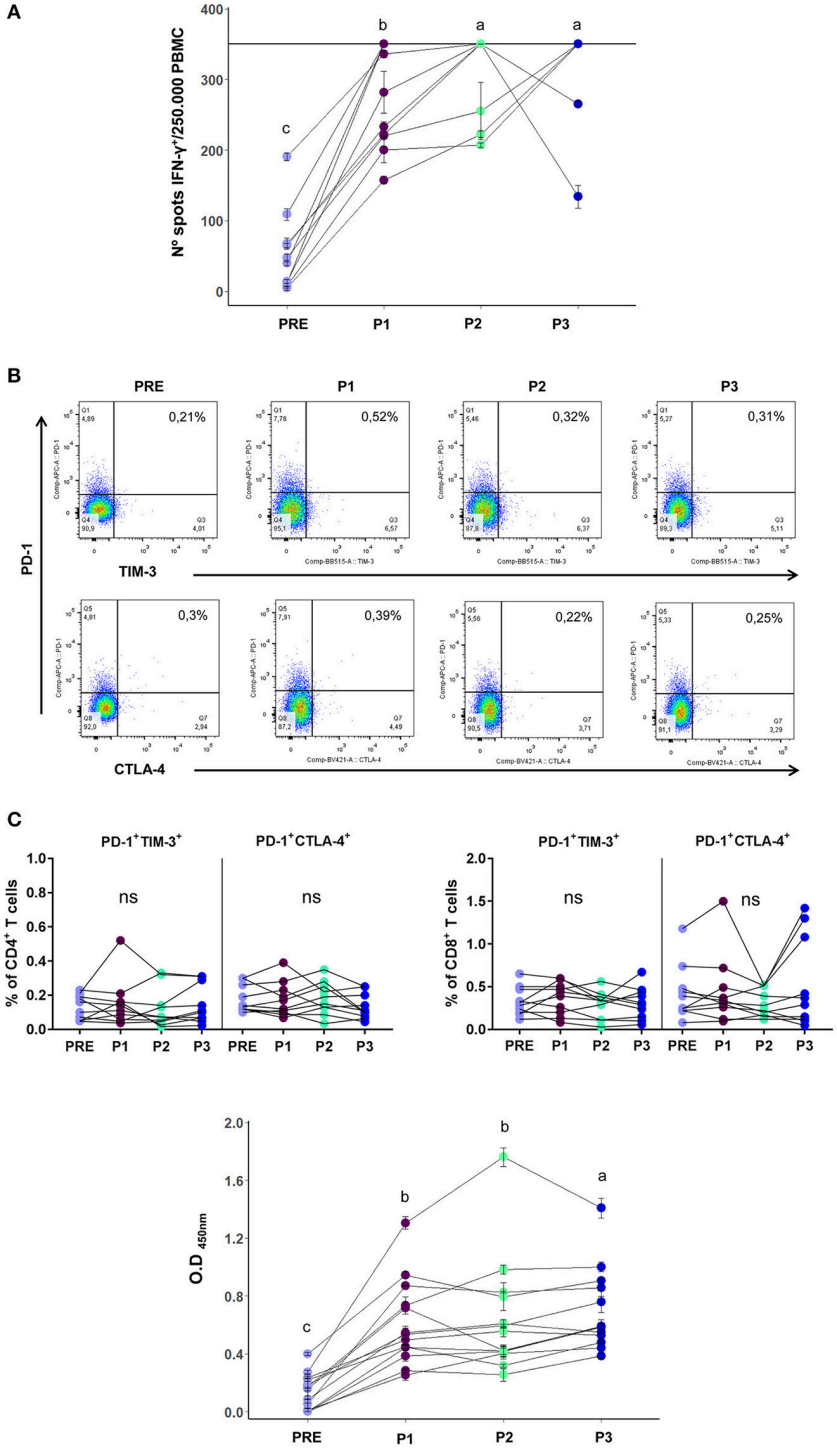
**(A)** T cell reactivity, as measured by ELISPOT, of PRE/P1/P2/P3 vaccinated patients samples against vaccine CSF-470 lysate, displayed as number of IFN-γ positive spots per 250,000 PBMCs (*n* = 10). The horizontal line in the graph at 350 spots indicates the highest number of spots that could be counted per well with this assay. Mean and standard deviation calculated from 2 replicates done in the same experiment, are shown for each sample. **(B)** Upper panel: Representative dot plot of inhibitory receptors PD1, TIM-3, and CTLA-4 co-expression analysis in CD4^+^ T cells. Lower panel: PD-1^+^TIM-3^+^ and PD-1^+^CTLA-4^+^ population frequencies in CD4^+^ and CD8^+^ T cells of PRE/P1/P2/P3 vaccinated-patient blood samples of (*n* = 10). **(C)** Serum antibodies from PRE/P1/P2/P3 vaccinated-patient blood samples that are directed against the melanoma cell lines composing the CSF-470 vaccine (1/10 serum dilution, *n* = 13). Mean and standard deviation calculated from 4 replicates done in the same experiment, are shown for each sample. Distinct letters represent statistically significant differences between means (*p* < 0.05).

In a previous Phase II study we demonstrated that after the first 6 months of treatment (P1 sample), at which point 5 vaccinations had been performed, T and B cell immune responses developed against the vaccine Ags (1). In the current study we extended the analysis to P2 and P3 samples to monitor long term immune responses induced by the vaccine. Out of the 19 vaccinated patients, 14 of them could complete the 2-year clinical trial. We found that the frequencies of CD4^+^ and CD8^+^ T cells remained unchanged throughout the entire treatment. On the other hand, the Natural Killer (NK) cell frequency that had showed a rise in P1 samples, returned to basal levels in P2 and P3 samples, as did regulatory T cells whose frequency had slightly decreased in P1 samples (7; Supplementary Figure 1). In a subset of 10 vaccinated patients that had completed the 2-year clinical trial, and from whom we had enough cryopreserved mononuclear cells from each time point (PRE/P1/P2/P3), we were able to complete the T cell response analysis after 24 months of treatment. The increased T cell reactivity against melanoma Ags observed in P1 samples remained high throughout treatment, as observed in Figure 1A. The ELISPOT assay was limited with a quantitative sensitivity of up to 350 spots, however we must note that some samples were qualitatively above this threshold. As we were also limited by the amount of patient PBMC samples collected, dilution analysis was not possible. These analyzed patients not only developed an immunological response to the vaccine, but also showed a positive clinical response since 10/10 were free of distant metastases at the end of treatment.

Since one of the most common features of exhausted T cells is the co-expression of inhibitory receptors (8), in the same subset of 10 vaccinated patients we performed the co-expression analysis of PD-1, CTLA-4, and TIM-3 in CD4^+^ and CD8^+^ T cells in PRE/P1/P2/ P3 blood samples. While we were able to detect stable expression of single exhaustion markers alone (Supplementary Figure 2), we show that successive vaccinations did not generate exhausted T cells since the co-expression of CD4^+^ and CD8^+^ PD1^+^ CTLA4^+^ and PD1^+^ TIM3^+^ populations remained low and with no changes during treatment (Figure 1B). It is important to note that the limited number of PBMCs available for analysis might have made it difficult to detect exhausted T cells, however supporting results are seen in the low levels of PD-1 expressing tumor infiltrating lymphocytes (TILs) observed in tumor biopsies from patient #006 as seen in Aris et al. (15).

Furthermore, the increase in serum antibodies against vaccine Ags observed in the P1 serum samples was maintained throughout the course of the treatment in 13 vaccinated patients, as it may be observed in Figure 1C; both the 1/10 and 1/100 dilutions showed an increase in serum antibodies (1/10 serum dilution is shown).

**Figure 2:**
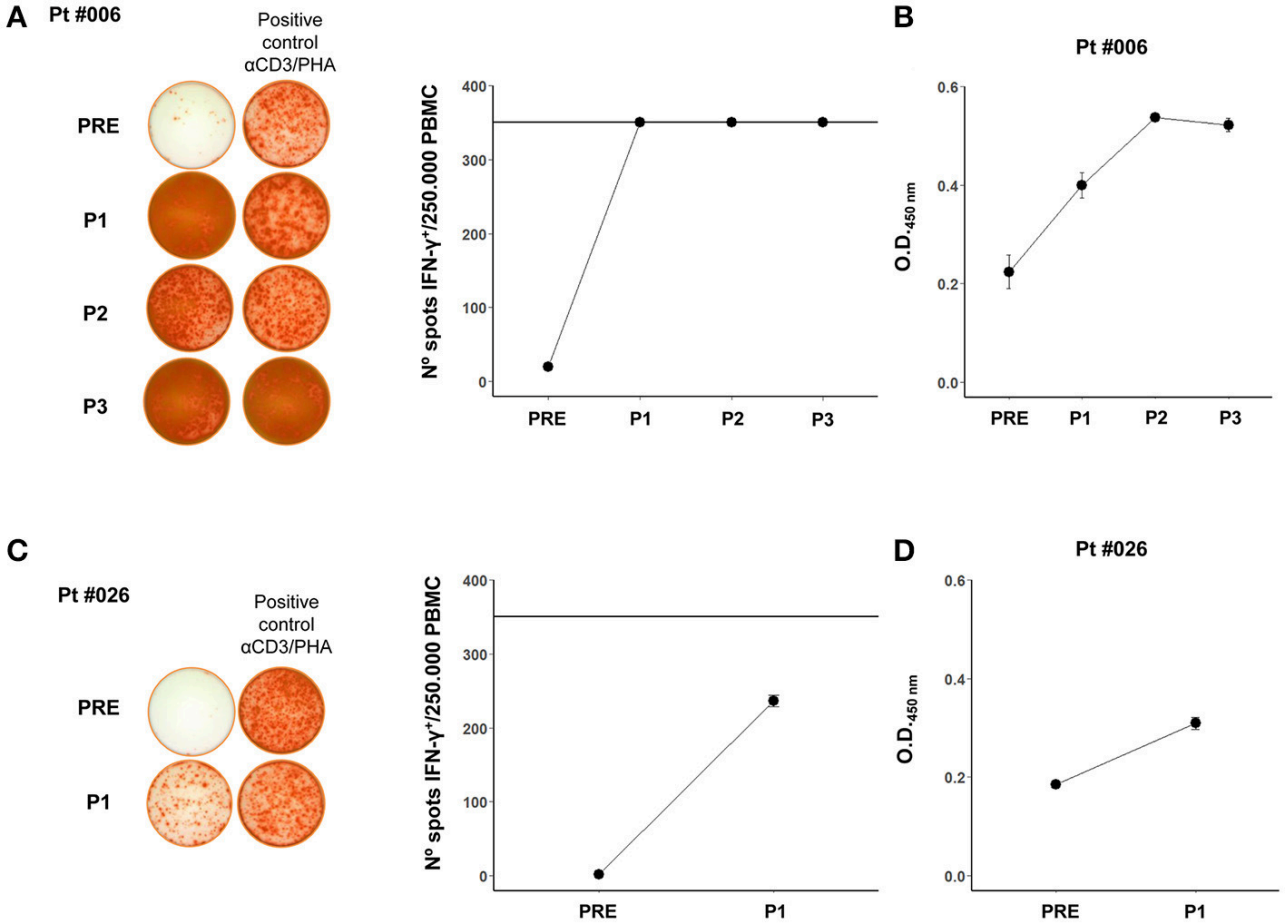
**(A)** Pt #006 T cell reactivity after 12 day stimulation with autologous tumor cell lysate displayed as the number of IFN-γ positive spots per 250,000 PBMCs. Representative images of spots for each mononuclear cell sample are shown. The positive control wells were restimulated with the monoclonal antibody against human CD3 OKT-3 and PHA (αCD3/PHA). The horizontal line in the graph at 350 spots indicates the highest number of spots that could be counted per well with this assay. **(B)** Pt #006 serum antibodies against autologous tumor cells (1/10 serum dilution), as shown in Mordoh et al. (1). **(C)** Pt #026 T cell reactivity after 12 day stimulation with autologous tumor cell lysate displayed as the number of IFN-γ positive spots per 250,000 PBMCs. The representative images of spots for each mononuclear cell sample are shown. The positive control wells were restimulated with αCD3 and PHA. The horizontal line in the graph at 350 spots indicates the highest number of spots that could be counted per well with this assay. **(D)** Pt #026 serum antibodies against autologous tumor cells (1/10 serum dilution). In all of the graphs, mean and standard deviation calculated from replicates done in the same experiment, are shown for each sample.

It was important to determine if the immune response against allogeneic vaccine Ags was also directed against the patients' own tumor Ags. This could only be determined in 2 patients (#006 and #026), in which primary cell cultures of tumor biopsies were obtained. In both patients, the T cell response, which was nihil at the beginning of treatment, increased after vaccination (Figures 2A,C) and a similar trend was observed for antibody formation (Figures 2B,D). However, the 2 patients had dissimilar evolution. Patient #006 recurred after the 2-year treatment with dermal and lung metastases. The dermal metastasis, from which the cell line was established, was highly infiltrated with CD8^+^ lymphocytes (15); she was successfully treated with BRAF inhibitors and is still alive 7 years after entering the protocol. Patient #026 had a rapidly growing tumor and died 14 months after entering the study; therefore only the 6-month (P1) responses could be evaluated.

### Short term systemic responses

**Figure 3:**
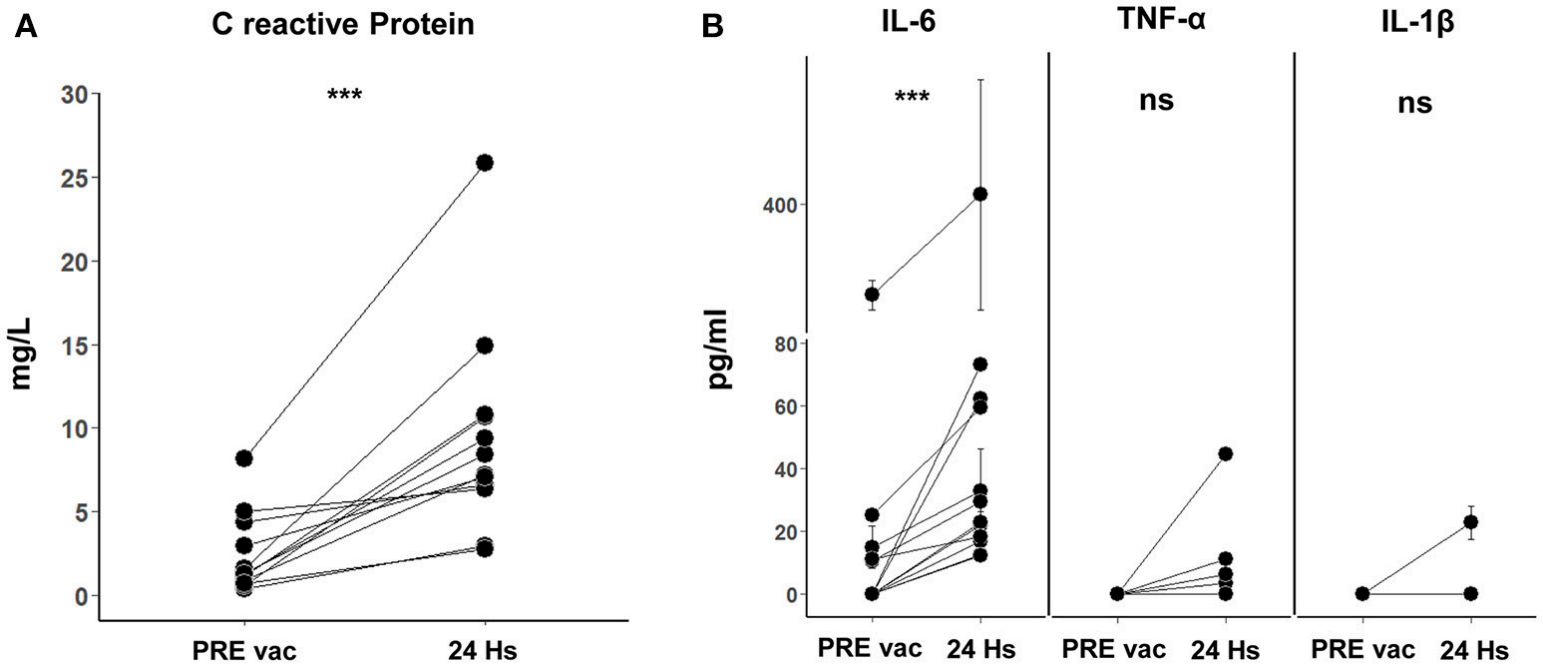
**(A)** C reactive protein (CRP) in 24 h post vaccination serum samples. Twelve samples of pre vaccination (PRE vac) and 24 h post vaccination were analyzed from 7 different patients. The normal physiological range for CRP is between 0 and 5 mg/L (Alexander Fleming Institute Laboratory of Clinical Analysis and Molecular Diagnosis, Buenos Aires, Argentina). A paired *T*-test was used to compare the means before and after vaccination of the paired samples (***p = 0.0005). **(B)** Pro-inflammatory cytokines IL-6, TNF-α, and IL-1β in PRE vac and 24 h post vaccination serum samples. For IL-6, 12 PRE vac-24 h post vaccination samples were analyzed for 7 different patients (Wilcoxon matched-pairs signed rank test was used to compare the paired samples, ***p = 0.0005). For TNF-α and IL-1β, 10 PRE vac and 24 h post vaccination samples were analyzed for 7 different patients (ns). Mean and standard deviation calculated from 3 replicates done in the same experiment are shown for each sample.

With the aim to comprehend the basis of the observed vaccine immune stimulation, we investigated whether vaccination with CSF-470 plus BCG and rhGM-CSF induced a short-term systemic response. For this purpose, in a subset of patients who continued vaccination beyond the 2-year protocol, we analyzed the presence of acute phase proteins and cytokines in peripheral blood samples taken pre and 24 h after vaccine administration. After analyzing 12 pre and 24 h post vaccination serum samples from 7 different patients, we found significant increases in concentrations of CRP (2.4 ± 2.4 to 9.4 ± 6.1 mg/l) and IL-6 (5.7 ± 8.7 pg/ml to 32.0 ± 22.0 pg/ml) (Figure 3A). In one patient (#022), a high serum IL-6 value was observed (as seen in Figure 3B) and thus excluded from the mean IL-6 values just reported; it should be mentioned that this patient had a large lipoma on his left shoulder and high levels of IL-6 were observed before entering the protocol which persisted throughout the duration as measured in P1, P2, and P3 samples (data not shown). As adipocytes are known to secrete IL-6 systemically, this comorbidity could be relevant (16). We found an increase of TNF-α only in 2/10 samples—and an increase in IL-1β only in 1/10 (Figure 3B). We could not detect IL-10 in these serum samples.

### IL-6 release *in vitro* by PBMC and CSF-470 cells

**Figure 4:**
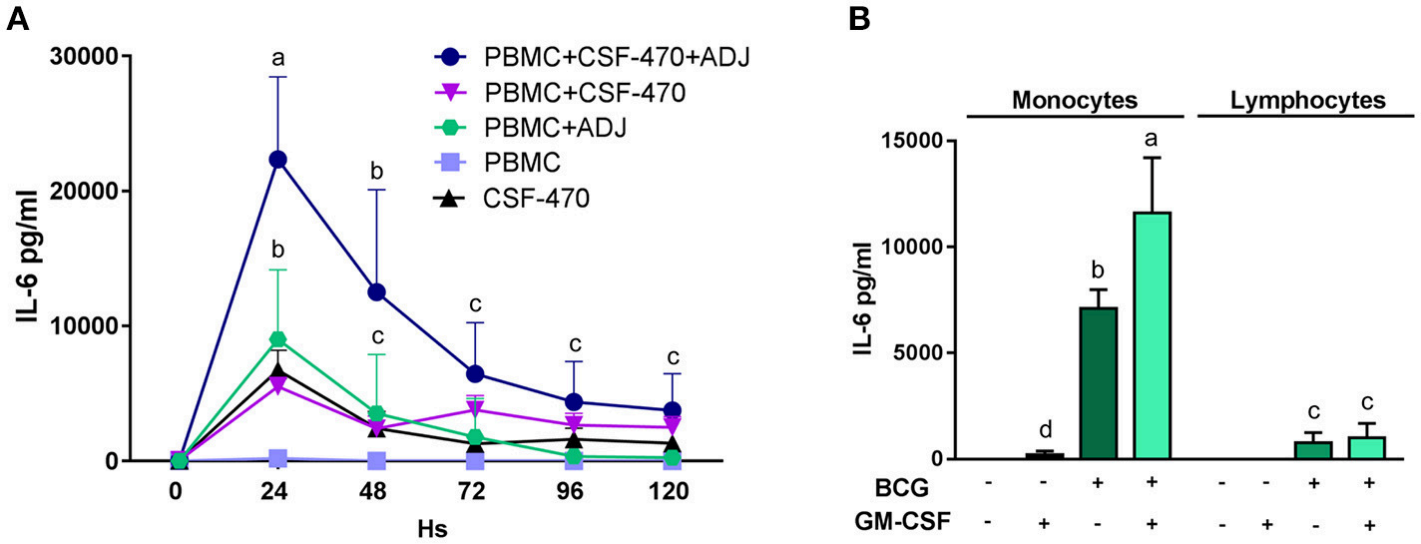
**(A)** Time course release of the IL-6 cytokine from cocultures of PBMC plus CSF-470 irradiated cells with or without adjuvants BCG and rhGM-CSF (ADJ), and CSF-470 irradiated cells alone (mean and standard deviation are shown, HD *n* = 4). Distinct letters represent statistically significant differences between means (*p* < 0.05). **(B)** IL-6 production from purified monocytes and lymphocytes stimulated with adjuvants BCG and rhGM-CSF for 24 h (mean and standard deviation are shown, HD *n* = 3). Distinct letters represent statistically significant differences between means (*p* < 0.05).

Since we observed that after vaccination most patients had increased blood levels of CRP and IL-6 to physiologically significant levels, we investigated the possibility that these proteins could be produced and released from the vaccination site. To study this, we cultured PBMCs with the various vaccine components and measured the concentrations of secreted proteins in the supernatants. With respect to CRP, we measured *in vitro* synthesis as described under Methods and could not detect any production (data not shown). It therefore may be assumed that CRP has a liver origin, as it has been extensively demonstrated (17). We then performed a time-course analysis of IL-6 release; this cytokine attained high levels, up to 22, 3 ± 6, 1 ng/ml, during the first 24 h of interaction between CSF-470 and adjuvants and then gradually subsided during the next 4 days; stimulated PBMC appeared to be its main producer (Figure 4A). As CSF-470 cells alone released moderate levels of IL-6, investigating the adjuvants BCG and rhGM-CSF as stimulators of PBMC purified into monocytes and lymphocytes revealed that monocytes are the main producers of IL-6 (Figure 4B). The adjuvant BCG appears to be the main stimulator, with the addition of rhGM-CSF acting synergistically to increase IL-6 stimulation as described before in Baqui et al. (18). Therefore, the systemic CRP detected may originate from the liver whereas IL-6 may derive at least partially from the vaccine injection site.

### CRP effect *in vitro*

**Figure 5:**
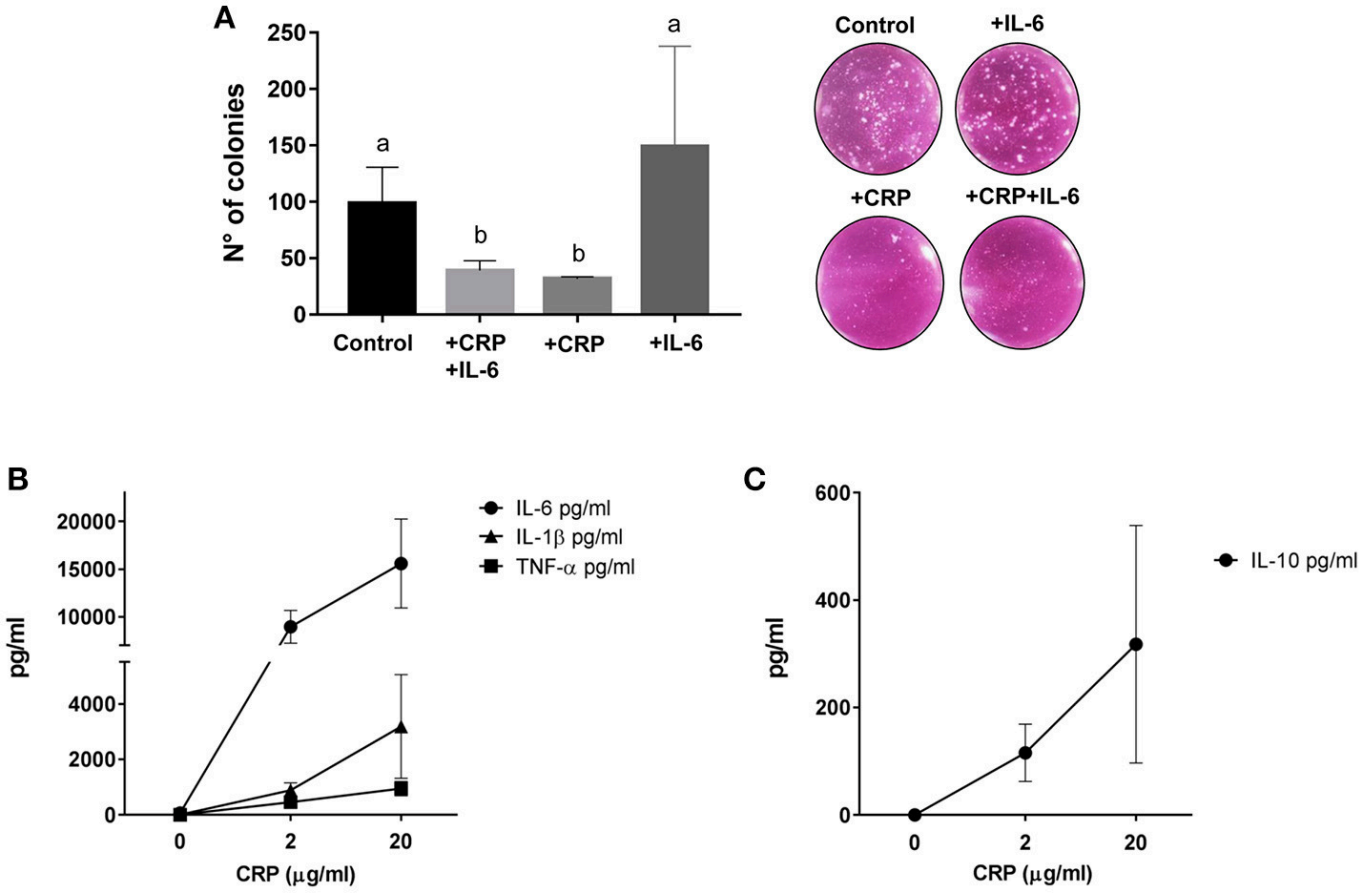
**(A)** Clonogenic assay performed on target MEL-XY3 cells after coculture with PBMC and fibroblasts with or without 20 μg/ml C reactive protein (CRP) and 100 pg/ml IL-6. Distinct letters represent statistically significant differences between means (HD, *n* = 3; *p* < 0.05). Representative photographs of colonies in each condition are shown. **(B)** Pro-inflammatory cytokines IL-6, TNF-α, and IL-1β released by PBMCs from HD stimulated with 2 or 20 μg/ml of CRP (HD, *n* = 3). **(C)** Anti-inflammatory cytokine IL-10 released by PBMCs from HD stimulated with 2 or 20 μg/ml of CRP (HD, *n* = 3). Mean and standard deviation are shown in all graphs.

Since CRP has been attributed the role of stimulator of the innate immune system, we then assayed *in vitro* if CRP enhances lysis of MEL-XY3 cells in the presence of PBMC. This cell line, one of the components of the CSF-470 vaccine, was chosen because it forms colonies in clonogenic assays. In order to liken the environment of a potential micrometastasis site, the tumor cells were cultured on top of a fibroblast monolayer in the presence of PBMCs with or without the augmented levels of CRP and IL-6 observed previously in patient sera. The results showed that in the presence of fibroblasts and PBMC CRP significantly inhibits colony formation by 65%; IL-6 appears to increase colony formation, although not significantly (Figure 5A).

Since circulating CRP was induced shortly after vaccination, we explored *in vitro* if CRP itself could induce the release of pro and anti- inflammatory cytokines. Using CRP levels comparable to those found in patient blood 24 h after vaccination we indeed saw a significant release of IL-6, TNF-α, IL-1β, and IL-10 from PBMC (Figures 5B,C).

## Discussion

In this paper we have extended our observations of the clinical study CASVAC-0401, and demonstrated that adaptive immunity is maintained during the 2-year vaccination period. Measuring the long term T cell response against the vaccine Ags by ELISPOT revealed that the number of IFN-γ secreting cells remained high and stable in most vaccinated patients. However, the fundamental question remains: is the T cell response also directed against the patient's tumor Ags? As there has been evidence demonstrating that IFN-γ increases melanoma cell lysis and Ag presentation of new epitopes through upregulation of immunoproteasome proteins (19, 20), it is reasonable to think that, in addition to the primary T cell response against the vaccine Ags, the successive CSF-470 vaccination scheme resulting in an increase of IFN-γ secreting T cells could also promote the presentation of novel, private Ags occurring in tumor microenvironment. With this in mind, here we were able to evaluate T cell reactivity against autologous tumor cells in 2 patients where primary cultures were established after recurrence either 2 years (patient #006) or 10 months after treatment (#026). This allowed us to show that patients' T cells not only reacted against the vaccine tumor cells but were also reactive against autologous tumor cells. However, it is not known whether the stimulated T cells are reacting against shared or private Ags, and future work includes elucidating the specificity of the reactive clones.

In the case of patient #006, a previous report from our group studied the clonality of T lymphocytes present in a subcutaneous metastasis (TILs) as well as those in blood throughout treatment. When looking at the top quartile TIL clones, which might be more relevant for antitumor function, pre-existing as well as late-emerging clones became prevalent, accounting for 51% of the total clones (15).

With respect to the persistence of humoral immunity in vaccinated patients, the same trend as observed in cellular immunity was found: Abs directed against both the vaccine and autologous (where available) Ags remained high, from the initial increase, throughout the vaccination period. Since we also demonstrated that successive vaccinations did not impact the co-expression of inhibitory receptors in T cells, we may assume that repeated CSF- 470 vaccinations stimulated a long term cellular and humoral immune response without generating exhaustion.

To further explore the mechanism of action of the vaccine CSF-470 we had previously established an *in vitro* system in which the different components of the vaccine were tested for their effect on PBMCs from HDs (21). BCG appeared to be the main inducer of the release of pro-inflammatory cytokines such as TNF-α and IL-1β by PBMC, coming mostly from innate immune cells. As it is known that such pro-inflammatory cytokines promote recruitment and infiltration of immune cells into the inflammatory site (22), BCG probably serves to help stimulate the early steps of the innate immune response occurring at the inoculation site. It has also been reported that vaccination with BCG induces the production of CRP, a member of the acute phase response proteins, that is mainly released by hepatocytes under the influx of IL-6 from inflammatory states (23, 24). When we analyzed the short—term systemic effect of vaccination in the serum of 7 patients, we observed that at 24 h after vaccination there was a significant increase in CRP and IL-6. Regarding the origin of these circulating proteins, our *in vitro* assays revealed that the combination of PBMC and CSF-470 vaccine plus adjuvants release large quantities of IL-6 peaking at 24 h, which suggests that the serum levels of IL-6 originate, at least in part, from the vaccine site. Whereas since in our *in vitro* model we did not observe CRP production, it is reasonable to infer that CRP is produced by hepatocytes after IL-6 release at the vaccination site.

IL-6 is a pleiotropic cytokine with a complex role in inflammation. IL-6 signaling has been shown to have both pro- and anti-inflammatory activity, initially directing the immune response to stimulate macrophages and T and B cell differentiation, and ultimately promoting resolution of inflammation (16). The classical cis signaling is restricted to hepatocytes and some leukocytes expressing the membrane bound receptor IL-6Rα; however, under inflammatory conditions a soluble form of the receptor sIL-6Rα is released into circulation which binds the ubiquitous gp130 signal transducer resulting in trans-signaling and greatly broadening the spectrum of IL-6 responsive cells (16). In contrast to classical evidence that IL-6 is pro-tumorigenic in the tumor microenvironment, it has more recently been shown that trans-signaling of IL-6 can contribute to anti-tumor adaptive immunity by guiding lymphocyte trafficking to lymph nodes and tumors, as well as supporting their activation, proliferation, and polarization to anti-tumorigenic phenotypes (25). The various actions of IL-6 are vast, yet importantly unchallenged is its role in stimulating the production of CRP from the liver and release into circulation.

CRP is a pentamer of 25 kDa monomers that binds phosphocholine (PC), phosphatidylethanolamine (PE), and other substrates such as exposed nuclear Ags through its recognition face, while binding Fcγ receptors and complement C1q through its effector face (26, 27). CRP acts as an opsonin that, like IL-6, has a range of both pro- and anti-inflammatory effects. In accordance, we demonstrated that CRP induced the release of both pro- and anti-inflammatory cytokines from PMBC.

The diverse roles of CRP are partly due to the existence of distinct isoforms and their different biological properties. Growing evidence suggests that the soluble pentameric CRP, which is measured in serum, irreversibly dissociates into monomers at sites of damage or inflammation, producing the bioactive monomeric form (mCRP). The mCRP appears to be the main pro-inflammatory isoform *in vivo* further triggering inflammation and increasing the recruitment and transmigration of phagocytes (28). In this study we demonstrated that PBMC killed tumor cells more efficiently when CRP was added to coculture *in vitro*. Although IL-6 appeared to play a slightly pro-tumorigenic role in these assays, the combination of CRP with IL-6 had a significant anti-tumorigenic effect. Since during cell damage there is often an exposure of CRP ligands on the outer membrane leaflet, and that in fact water-soluble PC and the major phospholipid PE are abundant in melanoma cells (29), it is therefore conceivable that CRP could bind tumor cell ligands and trigger the innate immune system activation. Supporting this, PE levels on the outer membrane leaflet are found to be augmented in stress conditions associated with tumor microenvironments (30). In a related context, the low pH found in tumor microenvironments and inflammatory sites has been shown to increase the binding affinity of CRP to certain ligands such as fibronectin, a protein found in abundance in tumors (31). Taken all together, the CRP enhancement of anti-tumorigenic lysis by PBMC and the potential for micrometastases to have favorable ligand exposure environments for CRP action, it is possible that increases in CRP in micrometastasis sites could contribute to the development of the anti-tumor memory T cell response through exposure of new Ags. Strikingly, we have shown *in vitro* that CRP also induces high amounts of IL-6 from PBMC and therefore it may be also assumed that a feed-back system sustaining CRP release could exist. It should be stressed, however, that patient #022 who had high serum levels of IL-6 even previous to vaccination did not show abnormally high CRP levels; some other regulatory mechanisms of CRP production may therefore exist. Considering that to our knowledge this is one of the first reports suggesting an antitumor effect of CRP, our results are significant and call for further investigation.

**Figure 6:**
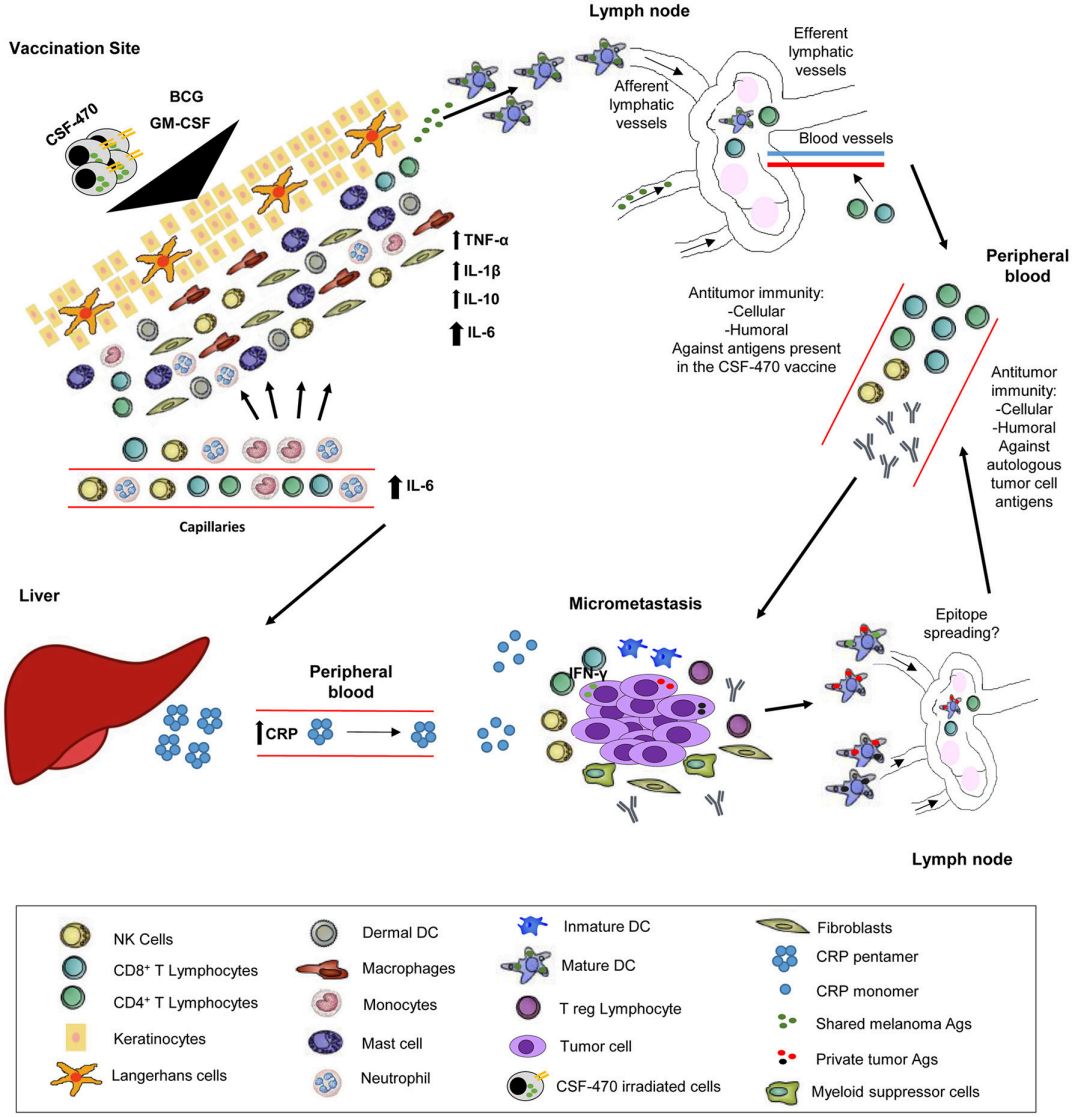
Proposed CSF-470 mechanism. After injection of CSF-470 plus adjuvants, a strong inflammatory reaction takes place, with release of pro-inflammatory cytokines and chemokines [20]. This reaction favors irradiated cell material to be incorporated and processed by Ag presenting cells, which then primes Naïve T cells in the lymph node. Subsequently, an antitumor cellular and humoral response is developed against tumor Ags present in the vaccine. Immune attack to micrometastases produces the release of antigenic material, which could contribute to epitope spreading, generating immunity against self-tumor Ags. After each vaccination, the release of IL-6 promotes CRP synthesis by liver cells. CRP acts in its monomeric form in the micrometastatic niche, stimulating immune cells and antibodies to attack tumor cells. This process, which takes place after every vaccination, could contribute to epitope spreading, and therefore, the augmentation of the immune response against self-tumor Ags.

In sum, we postulate that the interaction between the CSF-470 vaccine cells, BCG, and PBMC at the vaccination site cause the release of pro-inflammatory cytokines favorable for Ag presentation and activation of a T cell and antibody response directed against vaccine Ags. Additionally, during the few days after each successive vaccination, a CRP-mediated harmful activity of the innate immune system on melanoma cells could be triggered in micrometastatic deposits. Ensuing cell destruction would provide new waves of Ag exposure and presentation through Ag presenting cells, leading to a broader stimulation of the adaptive immune system, now targeting common and private Ags. This sequence of events could explain why repeated vaccinations with CSF-470, including BCG as an adjuvant, fosters an immune response directed against melanoma Ags. Hence, we would like to propose the model depicted in Figure 6.

In light of current advances in the field involving immune-checkpoint inhibitors and targeted therapies, research into incorporating the CSF-470 vaccine plus BCG and rhGM-CSF in combination with these adjuvant therapies is worth exploring and could yield high therapeutic value. With the idea that a higher number of tumor-reactive lymphocytes could be available to be further activated by anti-checkpoint antibodies, this could thus increase the benefit from adjuvant therapy in management of cutaneous melanoma.

## Ethics statement

This study was carried out in accordance with the recommendations of the Ethics Committee of the Instituto Alexander Fleming with written informed consent from all subjects. All subjects gave written informed consent in accordance with the Declaration of Helsinki. The protocol was approved by the Ethics Committee of the Instituto Alexander Fleming (Buenos Aires, Argentina), and by the local Regulatory Agency (ANMAT - Argentina). The Ethics Committee of the Instituto Alexander Fleming (Buenos Aires, Argentina) is reputed by the Central Ethics Committee of the City of Buenos Aires (Argentina).

## Author contributions

MP and HC: collection and assembly of data, data analysis and interpretation, and manuscript writing; GC: data analysis and interpretation; PB: sample collection and assembly of data; EL and MB: data analysis and interpretation and manuscript writing; JM: conception and design of the study, collection and assembly of data, data analysis and interpretation, and manuscript writing. JM was the principal investigator of the study CASVAC-0401.

### Conflict of interest statement

The authors declare that the research was conducted in the absence of any commercial or financial relationships that could be construed as a potential conflict of interest.

## Abbreviations

Ags: antigens
BCG: Bacillus Calmette–Guerin
CM: cutaneous melanoma
HD: healthy donor
PBMC: peripheral blood mononuclear cells
PBS: phosphate saline buffer
rhGM-CSF: recombinant human granulocyte macrophage colony-stimulating factor
CRP: C reactive protein
MAbs: monoclonal antibodies.
