# Supplementary figures and images for: Dissecting the Immune Stimulation Promoted by CSF-470 Vaccine Plus Adjuvants in Cutaneous Melanoma Patients: Long Term Antitumor Immunity and Short Term Release of Acute Inflammatory Reactants
Source: Front Immunol. 2018 Nov 2;9:2531. doi: 10.3389/fimmu.2018.02531 (PMC6224428; doi:10.3389/fimmu.2018.02531)

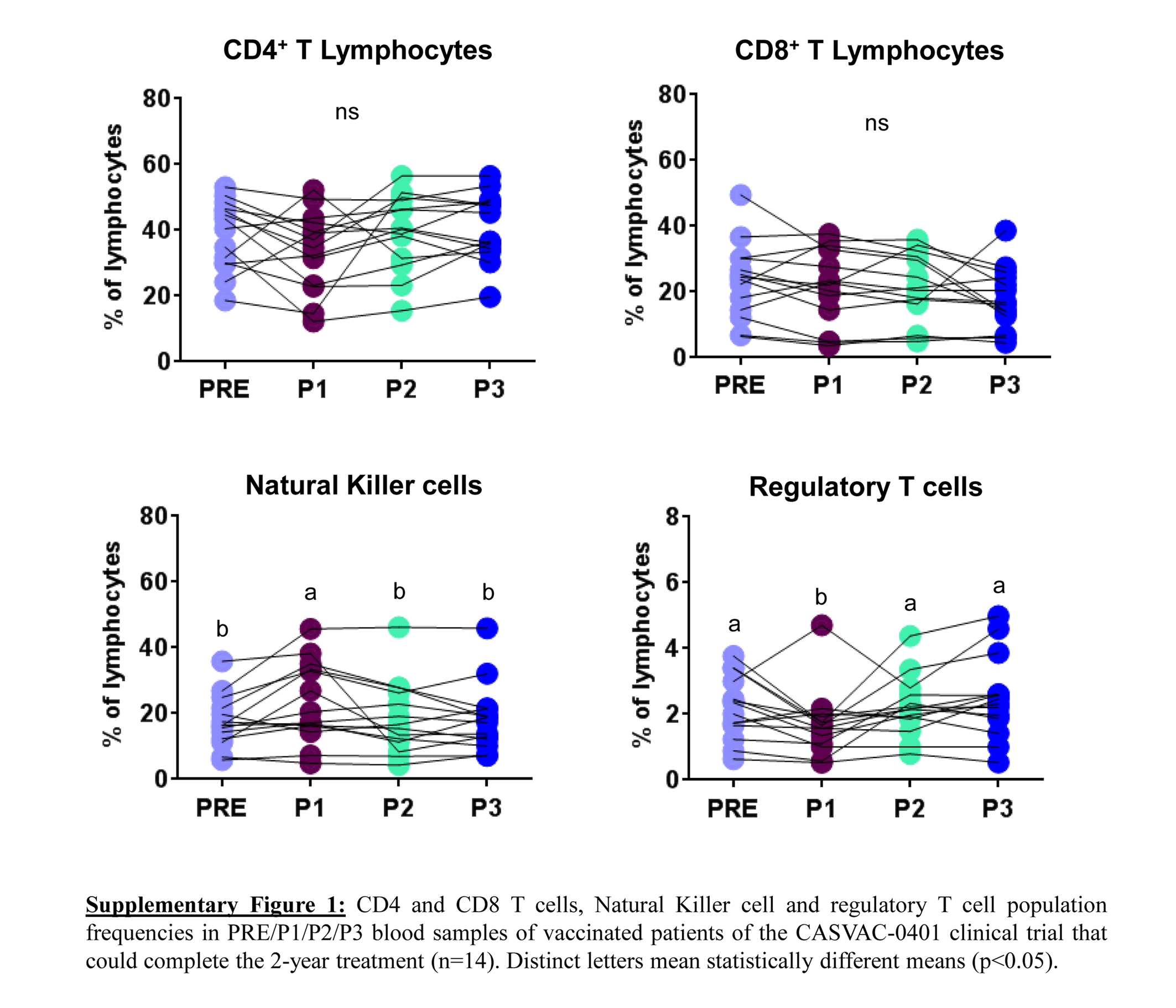

Supplement: Supplementary file 1 [file Image_1.TIF]

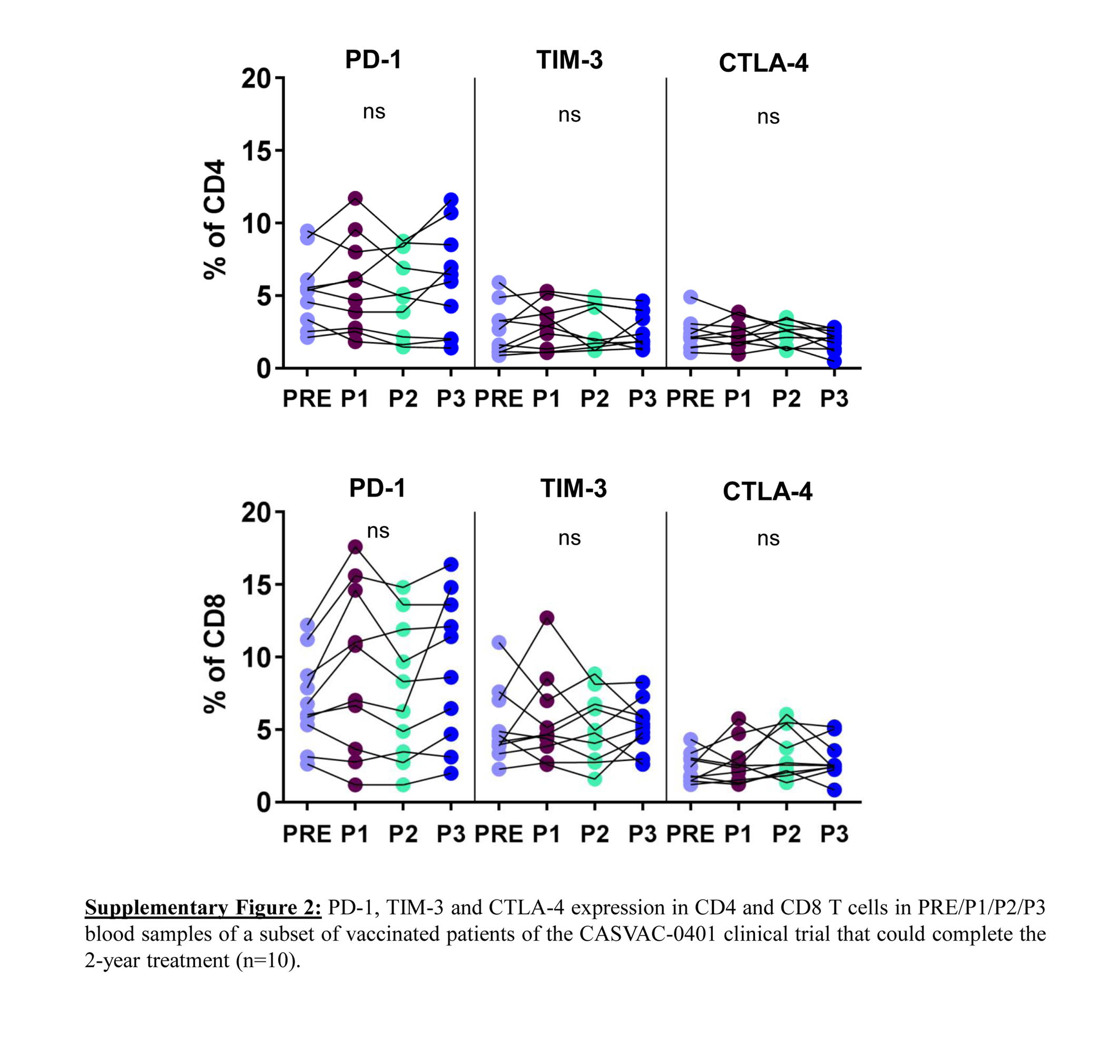

Supplement: Supplementary file 2 [file Image_2.TIF]
